# Use of nicotine substitute prescribed at hourly plus ab libitum intake or ad libitum for heavy smokers willing to quit: a randomized controlled trial

**DOI:** 10.1186/1747-597X-4-12

**Published:** 2009-06-02

**Authors:** Laurent Rey, Paul Vaucher, Françoise Secretan, Jean-Pierre Zellweger, Patrick Bodenmann

**Affiliations:** 1 Department of Ambulatory Care and Community Medicine, University of Lausanne, Switzerland

## Abstract

**Objective:**

To assess the impact of instructional guidance in the regular use of use nicotine nasal spray (NNS) on the true use of NNS during the first three weeks of smoking cessation for heavy smokers who are willing to quit.

**Methods:**

This randomized, open, controlled trial included 50 patients who were heavy smokers, were willing to quit, and attending an academic outpatient clinic in Western Switzerland. Patients were randomised to instruction on NNS use as "ad libitum" (administration whenever cravings appear; control group) or to use NNS when craving appears and at least every hour when awake (intervention group). Intakes were monitored using an electronic device fixed in the spray unit (MDILog™) during the first three weeks of use. Self reported abstinence from smoking at six months was confirmed by expired-air carbon monoxide. Using intention-to-treat analysis, random-effect GLS regression was used to calculate the mean difference of daily doses between groups controlling for lack of independence between measures from the same individual.

**Results:**

One patient was lost to follow-up. At baseline randomization, the group receiving instruction to use NNS hourly included more women, patients with previous desires to quit, and patients with more psychiatric comorbidities and less somatic complaints compared to the group instructed to use NNS with cravings (group imbalance). Both groups self-administered more than the daily recommended dosage of 8 uses. Mean daily usage was 13.6 dose/day and 11.1 dose/day for the group instructed to use NNS hourly and with cravings, respectively. Adjusting for baseline imbalance, the increased daily doses in the intervention group (hourly use) remained nonsignificant compared to ad libitum use (-0.5 dose/day; CI 95% -6.2; 5.3, from day 1 to day 7; and 2.3 dose/day; CI 95% -5.4; 10.0, from day 8 to day 21). Instructing patients to use the NNS daily had no effect on smoking cessation at six months (RR = 0.69; CI 95% 0.34; 1.39).

**Conclusion:**

Heavy smokers willing to quit use NNS frequently, regardless of the instructions given. Recommending the use of NNS only when craving appears for heavy smokers willing to quit seems acceptable compared to prescribing hourly administration.

**Trial registration-:**

ClinicalTrials.gov: NCT00861276

## Background

Cigarette smoking is currently the greatest preventable cause of death in our society; smoking cessation is regarded as one of the important preventive practices in modern medicine [1]. Use of nicotine replacement products increases the rate of smoking cessation 1.5 to 2 fold [2]. Nevertheless, there is uncertainty about the relationship between dose of replacement products, pattern of use, and success rate. Regarding the use of nicotine gum, improved outcome is reported with fixed dose rather than ad libitum usage [3]. Increasing the dose of nicotine replacement therapy has been shown to increase success rate [4]. Nicotine nasal spray (NNS) was developed to allow rapid delivery of nicotine through the nasal mucosa, allowing more rapid absorption than with the nicotine patch or gum. This rapid absorption and the user's ability to control the rate of use with NNS allow subjects to adjust the dose as needed. This type of nicotine substitution is particularly suitable for highly dependent smokers [5], willing to quit but with acute episodes of craving [6]. Different patterns of use have been observed between successful quitters and failures; successful quitters reportedly use higher doses [7]. Prescribing regularly scheduled use of NNS rather than instructing the patient to use the spray only when desired may improve the number of successful quitters. However, little is known regarding the response of patients to instructions for regular, scheduled use of nicotine substitutes. The aim of our study is to assess the effect of instructions for regular use of NNS on the true use of NNS during the first three weeks of attempted quitting; a secondary aim is to assess the effect of instruction for regular NNS use on smoking cessation success rates at 6 months compared with the currently recommended "ad libitum" use.

## Methods

### Participants

This open, randomized, controlled study was conducted in a group of 50 highly dependent smokers seen in the Department of Ambulatory care and Community Medicine in Lausanne, Switzerland. Patients were defined as highly dependent using Fagerstrom's criteria modified by Heatherton and al (smoking ≥ 20 cigarettes/day and/or smoking the first cigarette within 30 minutes after waking) [8]. To be included, patients had to be in the stage of preparation according to Prochaska and Di Clemente's stages of change model [9]. Exclusion criteria were a history of myocardial infarction in the preceding 3 months, pregnancy or breast-feeding, and use of any form of smokeless tobacco or other nicotine replacement therapy. At inclusion, demographic characteristics, smoking history, and nicotine dependence were recorded, and participants were asked about their smoking habits, prior quit attempts, current or prior psychiatric treatment, and motivation to quit. Subjects gave written informed consent to participate in the trial. The participants received advice to stop smoking completely at a fixed quit date. They were instructed how to use the NNS to enhance its acceptability and reduce local adverse effects.

### Randomization, allocation, blinding

Prior to data collection, a pharmacist prepared a randomization list of 50 blinded shuffled paper slips including 25 As and 25 Bs which were used to assign patients to treatment groups. Each paper slip was sealed in an opaque numbered envelope. Once a patient was included in the study and baseline data was collected, the sealed envelope was opened by the investigator to reveal the patient's allocation. Patients were blinded to the other intervention but were aware of their own. Investigator could not be blinded, as he was to give instructions on the use of NNS. During follow-up, the research nurse was not expressively made aware of the allocation but made all patients aware of the importance of using the spray when craving appeared. Statistician was blinded to which group received which intervention until the end of the analysis.

### Intervention

During the first month, subjects in the intervention group received instruction from the physician to use NNS when craving appears and at least 2 puffs/hour, for an average of 1 mg nicotine/hour when awake. Instructions were given to the patient as if this was the usual way of using the NNS.

### Control

In the control group, participants were instructed to use NNS as needed to suppress withdrawal symptoms when cravings appeared.

In both groups, 1) the use of NNS was free during the first two months, 2) during follow-up, physicians were trained to advise patients who experience craving to use the spray more often, 3) after one month, participants were advised to reduce the use of NNS if tolerable.

### Outcomes

During the first month, the number of puffs was recorded with an electronic device fixed on the spray unit (microswitch-actuated metered-dose inhaler chronology, MDILog™, model MDC-511, Medtrac, Denver, Colorado USA). The MDILog™ recorded the date and time of each activation. Only full days were taken into consideration; this means that the first day of use was not monitored. Doses are defined as grouped puffs that are spaced less than one minute apart. A series was defined as a sequence of puffs separated by more than one second but less than one minute. The normal number of puffs per dose should be two. Puffs that were monitored at the same second were counted as single.

Participants were informed of the aim of the MDILog™, but they did not see the results. Because the MDILog™ system showed variable reliability in previous studies [7], we followed a precise validation protocol to ensure data quality. Participants were asked to return their MDILog™ and all bottles of nicotine (empty, started, or full) at each visit. The research nurse checked every MDILog™ thoroughly with a software program and compared the total record of puffs since the last visit with the weight of the returned nasal spray bottles. If any technical deficiency or record discrepancy was detected, the MDILog™ was changed. Patients were monitored during the first month of use (a total of 27 to 35 days). Five patients had missing data completely at random due to the MDILog™'s initial technical failures. The trial steering committee therefore decided to extract data from the 21st full days of use instead of the initial 28 that were planned. All patients were instructed to administer two puffs for every dose. Data was collected to evaluate the number of series that had more or less than two puffs.

The criterion of abstinence was self-reported continuous abstinence from smoking from the beginning of the substitution to the end of the 6th month of follow-up, validated by an expired-air carbon monoxide (CO) concentration of less than 10 parts per million (ppm) at all visits (Bedfont Smokerlyzer, Bedfont Scientific Ltd., Rochester, UK). "Occasional slips" (i.e. less than 1 cigarette/day during the examined period) were tolerated. Smoking cessation was defined as successful in patients with smoking abstinence or "occasional slips" with a CO rate ≤ 10 ppm.

Follow-up visits were scheduled at 1 and 2 weeks and at 1, 2, 3, and 6 months. At each follow-up visit, participants were asked about cigarette consumption during the last 24 hours, smoking since last visit, and average number of puffs of NNS daily.

### Statistical analysis

The number of daily intakes was measured for each subject from both groups during the initial 21 days of monitoring (D+1 to D+22). Differences of daily intake between groups were computed using random-effect generalised least square regression (GLS) to take into account the lack of independence between measurements from the same participants. Interclass correlation coefficients were reported to estimate the proportion of variance related to individual characteristics. The analysis was stratified by week to see if differences between groups were constant through time and by weekdays. If important group imbalance was observed (>20% relative difference between groups), these factors were to be included secondarily in the regression analysis for adjustment.

Proportion of success were calculated for each group at 1 week, 2 weeks, 1 month, 2 months, 3 months, and 6 months with a 95% confidence interval (CI 95%). Relative risk (RR) of success at six months between groups was computed with CI 95%. P value was given for chances of a RR of 1 (chances that the intervention has the same effect as the control on smoking cessation).

Sample size was calculated for the primary objective only. Using data from a previous study [7], the expected average number of daily puffs (2 puffs = 1 dose) is expected to be 9 with a standard deviation (SD) of 8. The sample size was calculated to detect an increase up to 16 daily puffs (8 doses) with a significance level set at 0.05 and a power of 0.8. Each group was to include 21 participants. Expecting dropouts, the number of patients to be included was rounded to 25 per group. All calculations were performed with StataCorp. 2008 Statistical Software, Release 10.0 (College Station, Texas: Stata Corporation).

The study was approved by the ethical review committee for clinical research of the Department of Internal Medicine, University of Lausanne (Prot 29/99).

## Results

**Figure 1:**
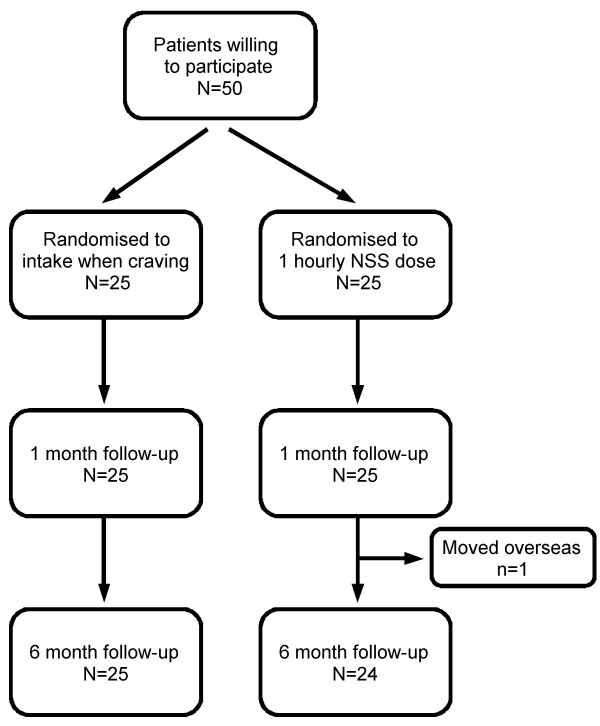
**Flow Chart**.

Data was collected from June 2000 to December 2001. As planned, 25 patients were included in each group. Four patients (two from each group) did not use their NNS from the second day on; two because of the side effects occurring at the testing session and two because they did not feel ready to quit smoking. These patients were included in the intention to treat analysis. One participant from the intervention group moved away and was lost to follow-up after his encounter at three months (Figure 1). Balance between groups was not achieved (Table 1). The intervention group included more females (40% vs. 16%), participants who had fewer previous attempts to quit smoking (2.2 vs. 3.1), had more co-occurring psychiatric disorders (44% vs. 24%), and fewer somatic complaints (56% vs. 70.1%) than the control group (Table 1).

**Table 1:** Patients' baseline characteristics.

	NNS 1 dose per hour/12 h a day	NNS Dose when cravings appear (control)
	n = 25	n = 25
Age		
Mean (SD)	40.2 (11.4)	40.8 (10.1)
Sex		
% male*	60%	84%
Education		
% high school/university	52%	58.3%
Health		
Psychiatric comorbidities*	44%	24%
Smoking habits		
Daily intake; mean cig/day (SD)	29.8 (11.4)	30 (10.3)
Baseline CO (ppm); mean (SD)	42.0 (16.1)	40.9 (19.0)
Years of consumption; mean (SD)	18.9 (11.0)	21.0 (9.0)
Previous attempts; mean (SD)*	2.2 (1.7)	3.1 (3.1)

*Over 20% relative difference between groups.

Cig: cigarettes; CO: carbon monoxide; SD: standard deviation.

**Figure 2:**
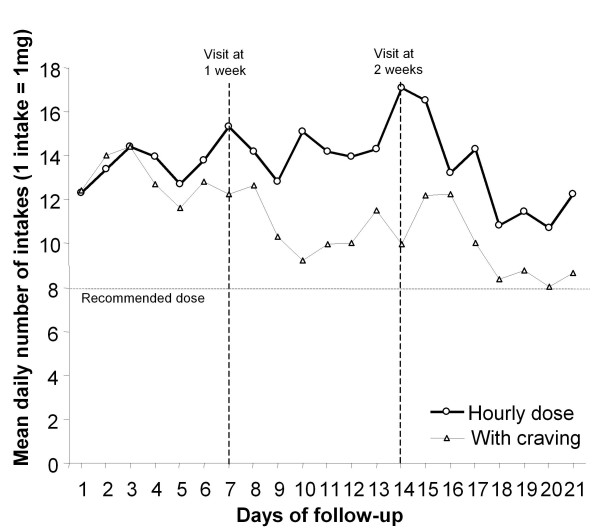
**Mean number of intakes taken daily for patients told to use NNS at least once every hour and for those told to use NSS every time cravings appear**.

NNS were used similarly between patients from both groups with important variances; these differences were due to individual characteristics rather than the intervention (Table 2). The calculated total number of 0.5 mg puffs highly correlated with the weighed consumption of NNS (r = 0.947; p < 0.001). After their physician visit, patients who were counselled to use the NNS hourly used the NNS an average of 13.6 times a day whereas those instructed to use it when cravings appeared used the NNS an average of 11.1 times per day; Figure 2 shows mean daily use of NNS. Patients told to use the NNS once every hour, 12 times a day, used an average 2.6 (CI95% -2.7; 7.9) more doses every day compared to those told to use the NNS when craving appeared; this difference was non significant. The fraction of variance due to individual characteristics other than of the prescribed intervention (instructed use of NNS) was 0.606. The difference in the number of daily doses between groups was less important during the first week (0.8; CI 95% -5.1; 6.7) than during the second (4.0; CI 95% -1.9; 9.9) and third week (3.0; CI 95% -2.5; 8.5). Including the patient's daily number of smoked cigarettes in the regression model did not improve the likelihood ratio of the model to predict the daily dose intake of NNS; the observed variance between patients cannot be explained by the patient's frequency of smoking before cessation.

**Table 2:** NNS use during the first 21 full days of treatment; data from 1050 days of observation from 50 patients.*

	NNS 1 dose per hour	NNS Ad libitum	Wald Chi^2 ^test	ICC^‡^
	Mean (CI 95%)	Mean (CI 95%)	p-value	ρ
**Doses**				
**Daily number of intakes**	**13.6 (4.7; 22.6)**	**11.1 (7.3; 14.8)**	**p = 0.332**	**ρ = 0.606**
Daily number of correct doses (2 puffs)	12.0 (3.9; 20.2)	9.3 (5.9; 12.7)	p = 0.263	ρ = 0.593
Daily number of single puff	0.5 (-0.4; 1.5)	0.8 (0.4; 1.2)	p = 0.470	ρ = 0.414
Daily number of doses over 2 puffs	1.1 (-0.1; 2.2)	1.0 (0.5;1.4)	p = 0.780	ρ = 0.331
**Period of use**				
Daily intake 1^st ^week	13.7 (3.6; 23.8)	12.9 (8.7; 17.1)	p = 0.788	ρ = 0.798
Daily intake 2^nd ^week	14.5 (4.4;24.6)	10.5 (6.3; 14.7)	p = 0.184	ρ = 0.696
Daily intake 3^rd ^week	12.8 (5.6; 22.2)	9.8 (5.8;13.7)	p = 0.287	ρ = 0.656
**Week days**				
Daily intake weekends	13.2 (4.3; 22.2)	9.7 (6.0;13.4)	p = 0.189	ρ = 0.525
Daily intake weekdays	13.8 (4.5; 23.1)	11.6 (7.7; 15.4)	p = 0.419	ρ = 0.652
**Use of NNS**				
Days NNS was used^†^	22.9 (19.0; 27.3)	23.0 (18.8;27.3)	p = 0.966	-
Total nicotine (mg)^†^	418 (294; 543)	296 (188; 405)	p = 0.131	-

* Results are drawn from random-effect GLS regression taking lack of independence from measurements from the same patients into consideration.

^† ^Individual level data was used for this analysis. T-test was used to calculate p-value.

‡ The Intraclass Correlation Coefficient (ρ) corresponds to the percentage of variance due to individual characteristics (cluster level).

Adjusting for group imbalance (sex, psychiatric comorbidities, number of previous desires to quit) and stratifying the analysis between day 1 to 7 and day 8 to 21, the observed increase in the number of daily doses was -0.5 (CI95% -6.2; 5.3) from day 1 to 7 and 2.3 (CI95% -5.4; 10.0) from day 8 to day 21.

**Figure 3:**
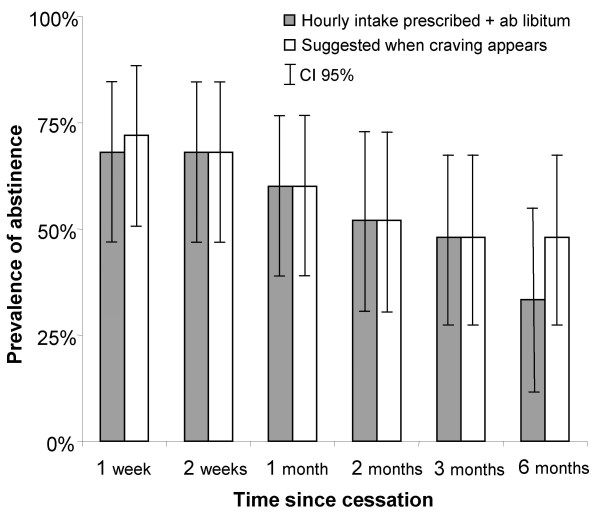
**Smoking abstinence during the 6 month follow-up (25 patients in each group)**. One patient in the intervention group was lost to follow-up after the 3 month visit.

The proportion of abstinent patients over time is shown in Figure 3. A total of 8 of 24 patients in the intervention arm and 12 of 25 patients in the control group successfully stopped smoking at 6 month follow up. Patients receiving instruction to use NNS once per hour had a lower cessation rate compared to those instructed to use the NNS when cravings appeared (relative risk of 0.69, CI95% 0.34; 1.39) although the difference was not significant (Fisher's exact test, p = 0.296). Eleven patients who had not ceased smoking reported a reduction in cigarette consumption (five in the intervention group and six from the control group). However, at six months only one patient smoked less than 50% of the initial amount while continuing to use the NNS.

## Discussion

This negative study shows that differences in NNS intake are more dependent on individual characteristics than on the recommended frequency of use. We observed frequent use of nasal spray in both groups. Average use was 13.6 intakes/day and 11.1 intakes/day in groups instructed to use NNS hourly versus with cravings, respectively. Nicotine doses were higher in both groups than those recommended by the manufacturer (8 daily intakes) and observed by Mabry et al [10]. Physicians were trained to follow patients and encourage them to use the NNS as much as possible to prevent craving and relapse. Furthermore, patients were all heavy smokers willing to quit, which could explain their regular use of the NNS independently of the given recommendation. Even if a slight trend towards more regular use in patients instructed to use the NNS hourly was observed, the minimal inferred average intake for the control group is clinically sufficient to justify the actual recommendation to use the substitute when craving appears. Seeing the patients regularly during the first two months could help increase compliance with the recommended use of NNS. We observed in both groups an increased use after visits with physicians. Feelings of empathy, encouragement in their initiative, and receiving counselling could help improve the regular use of NNS. Finally, differences exist in the use of NNS between individual patients. These differences were however not explained by the type of instructions given nor by previous smoking habits.

The proportion of success at 6 months (41%) in our study was slightly superior to rates seen in other studies using NNS, with reports varying between 10 and 35% at 6 months [5-7,11-14]. In our study, patients were followed regularly and counselling was offered to support their effort. Counselling and support might have increased use of the NNS, which in turn could have helped them in remaining abstinent [15]. Furthermore, positive instructions on the regular use of NNS given by physicians could have also helped in both groups by increasing expectations, which are known to have an important placebo effect [16]. A small risk of addiction to the NNS, due to the rapid absorption and high peak level, has been previously described [5,12]. However, the main reason for a prolonged use, as suggested by Hughes, is the fact that highly dependent smokers need a long transition period to recover from their physiological dependency [17,18]. In our study, only 1 patient among 50 was still using the NNS at six months. He was also the only patient who was smoking less than 50% of the initial amount after 6 months; all other patients who had reduced their consumption relapsed. This suggests that temporary reduction is probably real (no compensatory smoking) but not appropriate in the long term for highly dependent smokers [19].

To our knowledge, this is the second study where smokers have been instructed to use the NNS regularly and not on an "ad libitum" basis. As in our study, Tonnesen et al [14] did not observe a difference in the rate of success between smokers instructed to use the NNS regularly or on demand. Mooney et al [20] did not observe any improvement in the cessation rate at six months when encouraging use of nicotine gum with psychological interventions (brief feedbacks or contingency management). Nevertheless, in a large randomized trial, Shiffmann et al's observations support the theory of a causal relationship between higher use of nicotine substitute and success [4]. It therefore seems reasonable to believe that patient's use of substitutes depend more of their own reticence [21] or motivations [17] rather than instructions given by their practitioner.

The strength of this study is the precision in the measures of individual daily use of NNS using the MDILog™ and measuring effects of instructions in a pragmatic approach. The major limitation is the low power of this study. The small sample size resulted in clinical significant group imbalance for gender, co-occurring psychiatric disorders, and number of previous desires to quit. Nevertheless, controlling for imbalance in the regression analysis did not modify results. Our observations are limited to the use of the spray during the first month whereas most patients used the NNS for much longer. It cannot be excluded that the spray was used differently between groups after monitoring ended. This however had apparently no effect on the rate of success at six months. Finally, our observations concern heavy smokers willing to quit. Use of the NNS could differ for other levels of dependence or for smokers who are not willing to quit.

## Conclusion

Heavy smokers willing to quit use NNS in high doses, whatever the instructions given. Recommending the use of spray when craving appears is acceptable compared to prescribing fixed regular doses. For heavy dependant smokers willing to quit, when insisting on the use of NNS when craving appears, it does not seem to matter what instructions are given on the minimal recommended daily administration.

## Abbreviations

CI 95%: Confidence Interval of 95%; CO: Carbon monoxide; GLS: Generalised least square; NNS: Nicotine nasal spray.

## Competing interests

To the authors have no competing interests to declare. Pharmacia, Switzerland provided free NNS to the participants. They were not involved in data collection, the analysis of the results, in writing or correcting the manuscript, or in deciding whether the paper should be be published or not.

## Authors' contributions

LR, PB, and PV participated in the analysis and interpretation of data and drafting and revising the manuscript; LR, PB, JPZ, and FS participated in the conception and design of the study and reviewed the manuscript. PV and LR planned and analysed the data. All authors have read and approved the manuscript. They had full access to all of the data (including statistical reports and tables) in the study and can take responsibility for the integrity of the data and the accuracy of the data analysis.
